# Lessons learned from a virtual Community-Based Participatory Research project: prioritizing needs of people who have diabetes and experiences of homelessness to co-design a participatory action project

**DOI:** 10.1186/s40900-023-00456-z

**Published:** 2023-07-04

**Authors:** Saania Tariq, Eshleen K. Grewal, Roland Booth, B. Nat, Thami Ka-Caleni, Matt Larsen, Justin Lawson, Anna Whaley, Christine A. Walsh, David J. T. Campbell

**Affiliations:** 1 Department of Medicine, Cumming School of Medicine, University of Calgary, 3E33 CWPH Building, 3280 Hospital Drive NW, Calgary, AB T2N 4Z6 Canada; 2 Calgary Diabetes Advocacy Committee, Calgary, AB Canada; 3 Department of Community Health Sciences, Cumming School of Medicine, University of Calgary, Calgary, AB Canada; 4 Faculty of Social Work, University of Calgary, Calgary, AB Canada; 5 Department of Cardiac Sciences, Cumming School of Medicine, University of Calgary, Calgary, AB Canada

## Abstract

In Community-Based Participatory Research (CBPR), people with shared lived experiences (co-researchers) identify priority needs and work collaboratively to co-design an action-oriented research advocacy project. For this to occur, academic researchers must build mutually respectful partnerships with co-researchers by establishing trust. In the context of the COVID-19 pandemic, our objective was to virtually assemble a group of co-researchers (people with diverse but relevant experiences of homelessness and diabetes) and academic researchers who engaged in the CBPR process to identify a project that would address the difficulties of diabetes management while experiencing homelessness. Co-researchers were recruited to the committee from community homeless-serving organizations. Six co-researchers, one peer researcher and three academic researchers from Calgary, Alberta met virtually for bi-weekly committee meetings, from June 2021 to May 2022 to explore barriers to diabetes management and to complete a priority-setting exercise to determine the focus of our collective project. After reflecting on our virtual CBPR experience we present lessons learned related to: i) technical challenges and logistical considerations, ii) meeting virtually and building rapport, iii) driving engagement, and iv) challenges of transitioning from virtual to in-person meeting format. Overall, the process of conducting a CBPR project virtually to engage a group of co-researchers during a pandemic presents its challenges. However, a virtual CBPR project is feasible and can lead to meaningful experiences that benefit all group members, both from the community and academia.

**Supplementary Information:**

The online version contains supplementary material available at 10.1186/s40900-023-00456-z.

## Abstract (plain language summary)

In Community-Based Participatory Research (CBPR), we value peoples’ lived experiences as knowledge and believe that it can help in the design of research projects. In these projects, people with similar lived experiences work with researchers to design a research advocacy project that will make meaningful changes in their community. Trust and respect between all team members are essential for working well together. Establishing trust and respect can be difficult, especially when done virtually. We virtually convened a committee and completed a CBPR project. Our group consisted of seven people with lived experience of diabetes and homelessness and three academic researchers in Calgary, Alberta. We met every two weeks between June 2021 and May 2022 to explore possible topics for our CBPR project, which we narrowed down to their top priority. As we reflected on our experience of working together, we came up with four categories of lessons learned: i) technical challenges, ii) building rapport, iii) driving engagement, and iv) challenges of transitioning from virtual to in-person meeting format. While our experience presented its challenges, we found working virtually to create a CBPR project is possible and can be meaningful for all group members.

**Supplementary Information:**

The online version contains supplementary material available at 10.1186/s40900-023-00456-z.

## Background

The topic of working virtually and remotely quickly became critical during the early days of the COVID-19 pandemic as governments across the globe introduced social distancing restrictions [1, 2]. Moving online for everyday operations was difficult for many workplaces due to technological, logistical, and employee engagement challenges [3]. Since that time, numerous reports have focused on the benefits of virtual work, including the added flexibility for those with caregiving responsibilities and reduced barriers of commuting to a shared workplace [4]. A similar discussion of the advantages and challenges of remote work took place in the world of health and clinical research as social distancing restrictions were put into place [5, 6]. Academic research changed by needing to adapt recruitment strategies and data collection methods, as well as meeting ethical obligations and maintaining study rigor while shifting research processes online [6].

The discussion of virtual work is specifically salient for Community-Based Participatory Research (CBPR), which requires extensive trust and rapport to be built between academic researchers (individuals with formal research training in academia) and co-researchers (individuals with lived experience in the topic being studied, but who may have no previous research experience), and may include peer researchers (individuals with a dual role, who have relevant lived experience and formal research training) [7, 8]. In CBPR, the collaboration between academic researchers, co-researchers, and peer researchers is essential as all are equal partners through all steps of the research process [9]. By working in partnership to co-design action-oriented projects, CBPR often attempts to make relevant social changes in communities and tackle health inequities [10].

Since pandemic-related social distancing restrictions were implemented, several authors have published about the challenges of adapting CBPR projects to virtual spaces [11–14]. For example, a recent narrative scoping review found that challenges around virtual participatory methods included limited safe and reliable access to technology and venues that provided internet access, co-researchers' technological literacy, and maintaining physically distant social relationships [12]. Others have shared concerns regarding virtual CBPR approaches, including privacy issues and the ethicality of engaging socially disadvantaged populations, who faced significant social and economic burdens during the pandemic [15–18].

Between June 2021 and May 2022, we formed our team in a virtual context and began the initial steps of a CBPR project focused on addressing barriers to diabetes management faced by people experiencing homelessness. Diabetes is a complex condition which requires extensive self-management, including eating a diabetes-appropriate diet, taking medications as prescribed, participation in daily physical activity, and regularly visiting healthcare providers [19]. Experiencing homelessness can create numerous barriers to diabetes self-management, including limited access to healthy foods and medications, and other challenges that make self-management a low priority [20–22].

Our previous work in Toronto, Ontario, has shown that using a CBPR approach can empower people who have diabetes and have experienced homelessness [23]. By using a CBPR approach, we were able to help identify the most prominent barriers faced by people experiencing homelessness and co-design a relevant research-advocacy project that is meaningful to those with lived experience [20, 24]. However, this work was completed in person and used typical approaches to build trust and rapport, including sharing meals and a space where individuals could connect socially. As we moved to conduct a similar project in Calgary, Alberta, and understand the unique diabetes management challenges faced by people experiencing homelessness in this context, we were required to navigate pandemic-related social distancing restrictions. Guided by CBPR [25, 26], we aimed to form a committee of people with lived experience of diabetes and homelessness (i.e., co-researchers) who would guide all aspects of the research project, including setting a priority, identifying a research question and study design, data collection, analysis, and interpretation. The objective of this paper is to reflect on our team’s activities and lessons learned as we formed a group and determined an area of focus for a CBPR project using virtual teleconferencing with a group of co-researchers who have lived experiences of diabetes and homelessness in Calgary, Alberta. The activities which followed priority setting (i.e., co-design and implementation of a participatory action project) are currently ongoing and not included in this paper.

## Main text

### Activities

#### Forming the advisory committee remotely

Participants of our qualitative descriptive interview study investigating barriers to diabetes management while experiencing homelessness were invited to join the Calgary Diabetes Advocacy Committee (CDAC) for the CBPR study as co-researchers. Participants from the qualitative descriptive interview study were recruited from homeless-serving agencies in Calgary, Alberta who had experienced homelessness within the past 10 years. We used the Canadian Observatory on Homelessness’ definition of homelessness [27] and included those with any type or form of diabetes, which they were managing with medication and/or insulin (not those whose diabetes was only treated with dietary measures or physical activity). Existing contacts at agencies within the homeless-serving sector assisted with recruitment by putting posters up at their sites and by talking to clients who might be eligible. For those who contacted us, we began the process of enrolling them in the study by first obtaining verbal informed consent for participation in the research. All recruitment and interviews were done virtually due to the public gathering restrictions that were in place throughout the COVID-19 pandemic; the consent process and individual interviews were done over the phone.

A total of 14 individuals were interviewed over the telephone between May 2021 and February 2022. Characteristics of these 14 individuals can be found in Table 1. The interviews ranged from 28 to 68 min in duration. The academic researchers who completed the interviews included the research associate (EKG) and a graduate student (ST), who both brought experience and interest of working with people experiencing homelessness in CBPR or traditional clinical research projects. The peer researcher (ML)—an individual who has diabetes and lived experience of homelessness and had previously participated in the Toronto CBPR group [28]—also took part in conducting the interviews. The interviews and their analysis (not presented here) were overseen by the principal investigator (DJTC), an endocrinologist and health services researcher, who is interested in CBPR and had led the Toronto CBPR group [29].

**Table 1:** Demographics of people who completed an interview prior to joining the committee

Characteristics	Average or Count (n = 14)
Age (years)	51
Range	(27—65)
Gender (Women)	3
Diabetes type	
Type 1	3
Type 2	10
Other	1
Race	
Non-white*	3
White	11
Housing status (at the time of the interview)	
Rough sleeping or stable resident of a shelter	5
Stable private housing	4
Transitional housing	3
Stable community housing	2

^*^Non-white includes black and indigenous participants

Telephone interviews were conducted using a semi-structured interview guide (Additional file 1: Appendix A), which contained open-ended questions about the diagnosis of their diabetes, precursors to and precipitants of their housing instability, and their experiences with barriers and facilitators to diabetes management while unstably housed. The barriers to self-management while experiencing homelessness identified from these interviews included limited access to healthy foods, relevant diabetes education, and medications, and stigmatizing experiences with healthcare providers and shelter staff. Additionally, interviewees were asked about their general interest in joining the CBPR committee to generate a research advocacy project and their expectations for their involvement with a CBPR study.

After individuals completed the initial interviews, they were invited to join ongoing group meetings as co-researchers. Of the 14 individuals who completed the interviews, 10 individuals decided to join the CDAC. Those who did not join the CDAC provided various reasons for not joining, including full-time employment, a perceived lack of experience managing their diabetes while experiencing homelessness, or a lack of interest in the committee’s focus and goal. Some individuals did not provide a reason for declining to join the committee.

The study received ethics approval from the Conjoint Health Research Ethics Board at the University of Calgary (ID: REB20-0164). Participants in the interviews and individuals who went on to join the committee (i.e., co-researchers) received an hourly cash honorarium of $15 CAD for their participation.

#### Virtual committee meetings

**Fig. 1:**
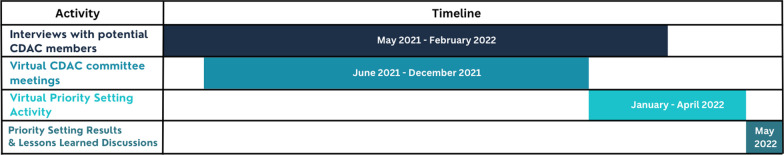
Timeline of the Calgary Diabetes Advocacy Committee formation and its activities

From June to December 2021 (Fig. 1), we held 20 one-hour-long discussions over Zoom™ (a video conferencing service) with an average attendance of four co-researchers, three academic researchers (a graduate student, a research associate, and the principal investigator), and a peer researcher. Zoom™ meetings were set up as recurring meetings every other Wednesday at 10 AM with the same meeting ID and passcode for each session to make it easy to remember and minimize confusion. After several meetings, we noticed that the timing was not working for some people due to work commitments and dislike of morning meetings, so the meeting was moved to 2 PM. Once the meeting time was changed, we noticed an immediate improvement in the level of attendance and engagement at group meetings. All co-researchers received a reminder text or e-mail (containing the date, time, and Zoom™ details) from the peer researcher a couple of days prior to the committee meeting.

To minimize the anticipated difficulties of online engagement [18] the research associate delivered tablets with unlimited data access, the Zoom™ app pre-installed, and meeting details preloaded in the app to the co-researchers a few weeks after their initial interviews and prior to their first committee meeting. The tablets provided to co-researchers were new and purchased from a large telecommunications provider in Canada who offered the best financial deal at the time for monthly data. These devices used the widely familiar Android Operating System™. Additionally, the teleconferencing platform used was recommended by our institution (Zoom™).

At the time of the physical exchange of the device, the research associate  demonstrated how to turn the device on, connect to Wi-Fi (if it was available), log-in to Zoom™, and turn the audio and video on and off, while following public health directives (e.g., maintaining a 2-m distance and masking). In addition to this orientation, there was a persistent need for ongoing training and re-orientation. At the beginning of each meeting, the academic researchers took a few minutes to explain and demonstrate basic functions to co-researchers so that all were comfortable using the device. We encouraged all to enable their device’s video function while keeping themselves muted except when speaking. The host of the meeting (research associate) retained the ability to mute individuals when needed if they forgot to do so themselves. One of the academic researchers was also available to provide IT support by calling individuals to walk them through the process of joining the meeting. While it took several sessions, eventually everyone in the group became quite familiar with these functions and teleconferencing meeting etiquette.

Meeting rooms opened 30 min prior to the scheduled meeting, which facilitated spontaneous discussions for those who chose to join early and allowed us to troubleshoot any video or audio issues. This extra time, in conjunction with planned ‘icebreaker’ activities during the meetings, contributed to building rapport between and amongst academic researchers and co-researchers. Committee meeting discussions were guided by a living 'terms of reference' document that contained guidelines and expectations of academic researchers and co-researchers’ commitment and engagement with the group, including compensation/honoraria, roles, and responsibilities. The ‘terms of reference’ was adapted by co-researchers in the first few sessions from a similar document used by the preceding Toronto-based group, and continually changed as we reviewed the document every couple of months.

Discussions during these meetings primarily focused on the barriers or facilitators of managing diabetes while experiencing homelessness, including accessing diabetes-appropriate foods, storing and accessing medication, and travelling to medical appointments. For the first couple of sessions, the academic researchers brainstormed possible topics of discussion based on the literature and broad barriers and facilitators that multiple co-researchers mentioned in their initial interviews. As the group continued to meet, co-researchers also brought up topics of interest, which were added to a ‘parking lot’ and revisited in future sessions. These topics were important to explore through discussion as a group, as we anticipated these discussions would build rapport between all attendees, increase the knowledge and understanding of academic researchers (who had no lived experience), and inform our group priority setting activity.

All discussions were co-facilitated by an academic researcher and the peer researcher, who often led discussions by sharing his own lived experience to encourage conversation and build rapport with the co-researchers.

In addition to these discussions, the principal investigator (who is an endocrinologist) led 15–30 min diabetes education sessions titled "Diabetes 101", which occurred every other session. The topics covered in these sessions were determined by co-researchers, who posed questions before or during the sessions about long- and short-term diabetes complications, treatments, and self-management principles. Topics were explored using lay terms and the whiteboard feature on Zoom™, which allowed illustration of various principles in a more engaging way than solely through discussion.

As co-researchers started attending the committee meetings regularly, we incorporated 30–60 min "Research 101" sessions, which introduced co-researchers to the research process and provided research skills training. The research associate and graduate student facilitated these workshop-style sessions by presenting PowerPoint presentations and leading interactive activities on the research process, CBPR, research question development, research ethics, recruitment, qualitative and quantitative methodologies, conducting qualitative interviews, and writing and interpreting research abstracts.

#### Virtual priority setting activity

After presenting possible priority-setting methodologies, the co-researchers voiced their preference for using a modified nominal group consensus process to determine the focus of the research advocacy project. The academic researchers facilitated the process, and only those with lived experience of diabetes and homelessness (i.e., the peer researcher and co-researchers) participated in the voting. The consensus process began by posing the question, “*What would make it easier for people experiencing homelessness to manage their diabetes?*” to help guide the co-researchers as they individually created lists of potential areas for improvement. Twenty-four distinct areas of improvement were identified from the individual lists generated. The committee then discussed, combined, and grouped the 24 items into eight larger categories over four virtual committee meetings to facilitate prioritization of the areas of improvement. The eight categories identified are presented in Table 2.

**Table 2:** Eight categories developed by co-researchers when answering the question “*What would make it easier for people who are experiencing homelessness to manage their diabetes?”*

Category	Areas of Improvement suggested by co-researchers
Diabetes awareness	Shelter staff receive training or education on diabetes; Normalizing insulin use in public
Dignity	Shelters creating a space where people feel respected and safe; Educate on equity and empathy
Access to medication	Let people access medication when needed; Create safe space that lets people take medication on time; Enhanced pharmaceutical insurance
Access to healthy foods	Work with dietitians to address diet needs in shelter; Increase nutritious food donations; Address diabetes special diet supplement amount from provincial disability insurance program
Diabetes education	Materials specifically created for people using shelter services; Education on medication, exercise, and diet
Screening for diabetes	Option to provide onsite A1C testing for people entering shelter system; Educating people to recognize signs of prediabetes
Access to diabetes-specific health services	Permanent diabetes clinic for those experiencing homelessness; Improve access to screening/testing facilities
Housing	Tackle the root causes and contributors of homelessness and explore housing solutions

**Fig. 2:**
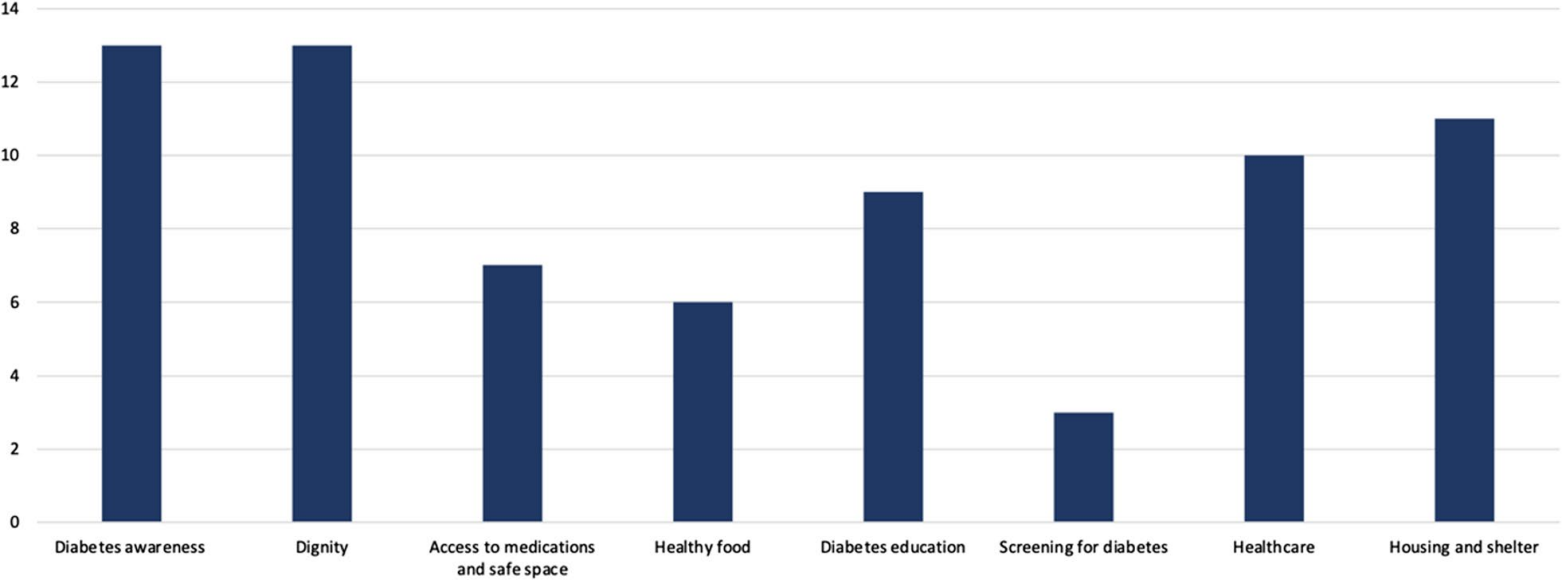
Number of points for eight key areas were added to give a final score when ranking areas of improvement identified by co-researchers

To determine key areas, we planned a virtual prioritization meeting in which the co-researchers and peer researcher consecutively ranked and then rated the categories, anonymously, using Zoom’s advance polling features. Results were shared immediately after each activity to allow time and space for co-researchers and the peer researcher to discuss their combined choices. Co-researchers and the peer researcher were first asked to rank-order their top four out of the eight categories generated using the ‘matching’ poll feature. Four points were allocated to the top choice, 3 points to second choice, 2 points to third choice, and 1 point to fourth choice. In this exercise, Diabetes Awareness and Dignity were ranked as the group's highest priorities, each scoring 13 points (Fig. 2).

**Fig. 3:**
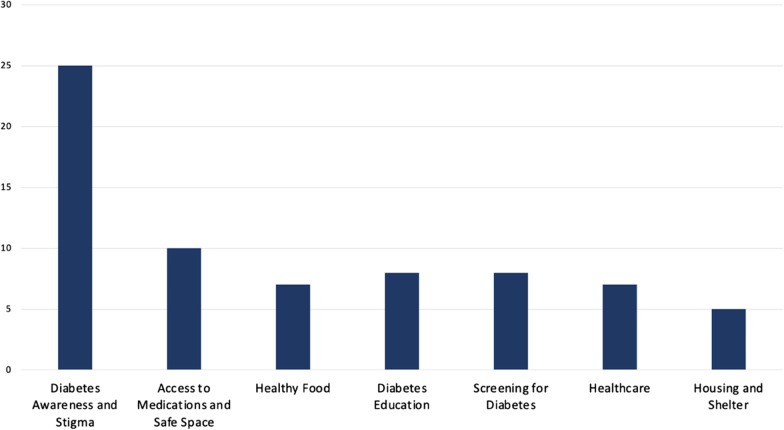
Points assigned in any amount in rating the seven areas of improvement identified by co-researchers

As the committee discussed the results, a few co-researchers highlighted the difficulties they had in distinguishing between the categories of ‘Diabetes Awareness’ and ‘Dignity’ and as a group they decided to combine them for the subsequent rating activity into ‘Diabetes Awareness and Stigma’. The committee suggested that increasing awareness about diabetes among shelter staff, health care providers, or others would lead to changes in services and reduced experiences of shame or stigma, thus increasing dignity. For the rating activity, the co-researchers and the peer researcher were instructed to assign 10 points to the categories of their choosing, in any denomination, using the short answer feature on Zoom™. To minimize issues with assigning too few or too many points, we presented examples of the rating exercise using favourite ice cream flavours. Diabetes Awareness and Stigma remained by far the group's highest priority, with 25 points allocated (Fig. 3).

### Lessons learned

Despite working on previous CBPR projects with people who have experienced homelessness [23, 30], the academic researchers had never done so in a virtual setting. We secured funding for this project in the spring of 2020, just as the pandemic was starting to end in-person interactions. Due to the importance of meeting communities 'where they are at,' we deemed that it was not possible or feasible to do this work, leading us to place this project on hold [31]. However, in the spring of 2021, we felt we needed to start the project to be accountable to our funder and because of the imperative to generate knowledge on the struggles of this population in the midst of the pandemic. We were initially unsure how to begin this process in the context of pandemic restrictions. Fortunately, one research team member (CW) had already done virtual CBPR work with other groups [32, 33] and she recommended that we proceed in this fashion. We, therefore, created a plan to eliminate potential barriers by providing internet-enabled tablets with technology and training to co-researchers and attempt to build rapport through online CBPR-related activities. We formed four lessons learned we believe to be relevant for future researchers and communities to consider when working on virtual CBPR projects with socially disadvantaged populations by reflecting on our activities over multiple group meetings with academic researchers, a peer researcher, and co-researchers.

#### Rigor

Trustworthiness of our reflections was enhanced through multiple efforts. This paper was informed by CBPR principles [34], as the aim and content (i.e., lessons learned) of the manuscript were developed collaboratively over multiple group meetings. This improved credibility, as we repeatedly met over multiple meetings to brainstorm, develop, and review the lessons learned shared in this paper. Dependability of our work was enhanced by maintaining detailed meeting minutes of these committee meetings. The confirmability of our reflections was strengthened by involving multiple perspectives from different investigators (co-researchers, peer researchers, and academic researchers) who had unique expertise and interests. Co-researchers and the peer researcher, (RB, NB, TK, JK, AW, ML) were involved in the writing process and provided multiple rounds of edits to the initial draft of this manuscript, which was written by the academic researchers (ST, EKG, DJTC). As a result, co-authorship was given to all parties who met criteria for inclusion.

#### Technical challenges and logistical considerations should be anticipated

Successfully using technology was a daunting hurdle for our CBPR project. Many in the academic and business worlds faced similar anxieties but became familiar with teleconferencing software for virtual meetings over the pandemic-affected year prior to our study's launch [3]. However, many co-researchers did not have the same exposure or opportunities to use teleconferencing software, including Zoom™, leading to numerous technology-related challenges at the beginning of this project. For some, these issues were frequent and frustrating, requiring technical support from the academic researchers. In some cases, the technical challenges and the difficulty of resolving them over the phone led some group members to reconsider participating in the project and eventually drop out altogether. For these individuals, the academic researchers followed up and brainstormed with the individual of alternative ways they would be able to continue participating (i.e., calling in, joining from a public space). However, with further discussion, these individuals shared other reasons why they did not want to further attend meetings, including increasing frequency of medical appointments and/or an overall lack of interest in the group and the discussions. For those co-researchers who continued in the committee, they learned how to use the technology quickly and had very few technical problems after a few meetings. For example, we noticed that although we had not taught the co-researchers how to use the Zoom™ chat feature, they had figured out how to message each other to share health and social service resources with one another.

Another logistical challenge we faced was related to the private spaces and location people used to join the meetings. At the time of the study, most of the co-researchers were currently residing in private or supportive housing arrangements. Joining meetings was simpler for those with a private space as they could do so with fewer challenges than those in shared living spaces, who struggled with including respecting others’ privacy and finding a quiet space to join. We had less success engaging people actively experiencing more acute housing instability, such as those who were staying in emergency shelters or sleeping rough, despite our tablets being internet-enabled through cellular networks to account for the anticipated challenges for those in this situation. To support co-researchers facing this challenge, we followed up with phone calls to brainstorm possible solutions that were feasible and appropriate for their unique situation. For example, one co-researcher who was residing in an emergency shelter found it too noisy and distracting to join meetings from the shelter, so for group meetings we found out he could join meetings from the public library once it had reopened. For another co-researcher, we found headphones that would provide them with the desired level of privacy when joining the group meetings.

Future CBPR teams should consider the time and effort required to set up the virtual meeting space and recognize the comfort level of co-researchers with technology. For our virtual meetings to begin, significant planning was needed to ensure co-researchers had access to internet-connected tablets and that they could join Zoom™ for the committee's reoccurring meetings. Overall, we found the logistical efforts required of the research team in a virtual setting was similar to what is required for in-person CBPR activities. The time that would have been spent on travelling and picking up food was spent on preparing and troubleshooting issues with Zoom™. Furthermore, for people who have lived experience of homelessness, finding a location to join the meeting was a time-consuming effort. Commute time was only saved for those with secure, private housing, as those currently in the shelter system travelled to quieter public spaces to join the meeting. While doing the research virtually enables researchers to engage and do research with people where they were not physically present, this still required the study team to connect with them in-person to transfer the tablets to them and train them on how to use the teleconferencing software. It is important to consider that the experiences of co-researchers may have been different if we used different hardware (e.g., laptops), operating system (e.g. apple OS™), and/or software (e.g., Google Meets™, MS Teams™).

#### Building rapport virtually is challenging but imperative

Once we had addressed our group's technical and logistical issues, we attempted to connect with the co-researchers. One of the cornerstones of CBPR is the academic researchers' ability to build rapport with the group [35]. Rapport building is a challenging feat at the best of times, made yet more complicated by interacting exclusively on virtual platforms. One of our major assets in this study was the peer researcher (ML) who was able to reach out to the co-researchers and move discussions forward by sharing his own experiences to put the co-researchers at ease and create an environment where all felt safe enough to share their own experiences. Due to his lived experience and understanding of the specific needs of the co-researchers, he could generate and build rapport with the co-researchers much more quickly than the academic researchers.

Future teams should consider involving a peer researcher or an individual who bridges the academic world and the community through their lived experience and training. However, the peer researcher did not remove all the barriers to building rapport, as there were other considerations beyond the shared lived experience of diabetes and homelessness. For example, some co-researchers discussed how they did not initially connect with anyone in the group, or the topics discussed, as they had type 1 diabetes instead of type 2 diabetes. Hence, having multiple peer researchers with diverse lived experiences involved at the initial stages of the CBPR project could help build rapport with all co-researchers. As the co-researchers become established members of the committee, they each begin to be able to take on the peer researcher role for newer committee members who join subsequently.

Additionally, peer researchers must consistently consider their roles and responsibilities in each CBPR activity. Previous work has highlighted how the dual roles played by peer researchers can lead to confusion and burnout for them [36]. To mitigate these possible issues, the PI, research associate, and graduate student regularly clarified the team members’ roles and responsibilities and met often with the peer researcher to debrief.

For academic researchers who have no lived experience of the research area and are looking to build rapport with co-researchers in a virtual setting, we found being engaged during conversations, using humour, and sharing knowledge and meaningful life experiences or information about yourself were all important. While planned ‘icebreaker’ style questions helped us get to know each other in a virtual setting (i.e., tell the group your name and then whether you would describe yourself as a dog person, or a cat person, and why?), opening the Zoom ™ meeting room before the scheduled meeting started was the most helpful as it minimized the formality and pressure of a scheduled meeting and mimicked the usual socialization time before or after an in-person meeting. However, everyone did not use this earlier time, sometimes joining meetings late and only after they were sent a reminder text or phone call. Additionally, remaining engaged during our conversation was not always easy as we found we were all easily distracted by other tasks. For instance, during the meeting the academic researchers occasionally sent emails and co-researchers occasionally ran errands or helped others in their physical proximity in crisis situations. The issue of multitasking remained throughout our virtual engagement and only stopped once we moved in-person after the priority-setting activity.

#### Driving engagement takes dedicated effort and may require multiple different approaches

CBPR requires a longitudinal commitment from academic researchers and co-researchers to be effective in its objectives [37]. Therefore, from our earliest interactions with potential committee members and in the terms of reference document, it was made clear that this committee was a long-term regular commitment. Among those who formally enrolled in the group, several have expressed their desire to seeing the process through, given their investment of time and effort. That said, it is important for the academic researchers to intentionally plan sessions to maintain high commitment levels to establish a priority and move on to co-designing a participatory action research project. In our virtual process, we faced problems with group fatigue and active participation as time passed. Even though co-researchers approached meetings with enthusiasm at the outset, everyone noticed that their interest seemed to wane, and a sort of fatigue set in over time. Over the course of the pandemic, ‘Zoom fatigue’ has been commonly experienced across many different virtual workplace settings [38]. Several co-researchers stopped attending meetings, decided not to continue with the group, or began turning off their cameras during meetings. The academic researchers initially thought that providing group members with assignments to complete between group sessions would strengthen their commitment. However, it had a paradoxical effect of reducing engagement among those who had negative experiences in formal educational settings and felt stigmatized by the expectations of ‘homework’.

After this failed attempt of engaging the co-researchers, the academic researchers asked for the group’s feedback on their level of engagement and why individuals were not attending as frequently. We heard the feedback that group meetings had become somewhat repetitive. Even though we discussed different topics each session, co-researchers were ready for something different. They were particularly motivated by the idea of helping others and contributing in a tangible way rather than continuing with virtual discussions. In response to the co-researchers request, we started introducing more frequent Research 101 and Diabetes 101 sessions to build capacity as a means to prepare them for the research advocacy project. Throughout the process of Research 101, the group became more comfortable with the idea of research. Additionally, we regularly reminded co-researchers that we were working towards the priority setting activity which would help us identify an area of change that would be the focus of our action-oriented research project. Future groups conducting CBPR virtually should consider collaboratively establishing a flexible timeline for changing shared goals and utilize capacity building activities to engage the group, understanding that group objectives and interests can and often do change over time.

#### Anticipate challenges when transitioning from virtual meetings to in-person ones

While we attempted to increase engagement, the committee’s fatigue continued. So, when the public health guidelines around the COVID-19 pandemic permitted and shared meeting places (such as campuses and libraries) began to open up, we held a couple of group and individual discussions with the co-researchers to determine if and when they wanted to move in-person. During these discussions we followed current public health guidelines around the COVID-19 pandemic (e.g., masking and keeping a 2 meter distance), and considered everyone’s comfort with meeting in shared space, and the feasibility of organizing and attending an in-person meeting. The groups’ unanimous decision resulting from our discussion was to move in-person immediately.

There were some difficulties that occurred after transitioning to in-person meetings. Namely, there was some awkwardness, especially for the peer researcher, who found the hybrid set-up challenging as he was residing in a different city and continued to communicate with the in-person group through Zoom ™^.^ Additionally, finding a location where we would meet was challenging. We decided to have the meetings at the library that was downtown. However, we quickly learned that some people had been banned from the space and others felt that some rooms were overly “sterile”, “hospital-like”, or “a petri dish” – due to the large windows in the conference rooms. We also considered one of the university’s downtown spaces but the committee told us that security features like the need to sign in and provide names, contact information, and signatures would deter co-researchers from attending the meetings. We also considered using spaces in shelters, but most people were not comfortable going back to emergency shelter spaces once they had become housed. Considering the various issues with each location, the groups felt the best option was the public library downtown, as there were meeting rooms that felt less “clinical” and the ban placed on some individuals was scheduled to end prior to the first in-person meeting. Most importantly, the library was easily accessible by foot or transit for everyone and did not require individuals to sign in.

The growth and collaboration that has occurred while working in-person far exceeds our virtual attempt. Being present in one physical space made it easier for people to stay engaged, not be distracted, and to socialize more with one another. Some co-researchers described the in person move helped them see the academic researchers as “real people” and not just "boxes on a screen”. Through the in-person conversations, we have been able to share and learn novel things about each other that had not been disclosed in our virtual discussions. Attendance also improved with bus tickets, coffee, food, and the opportunity to socialize. The majority of the co-researchers began to show up early to socialize with one another and participated in more lively discussions than our virtual meetings, leading us to extend our meetings from 1 to 1.5 hours and ultimately to 2 hours in duration. Additionally, as we moved in-person, other opportunities became available to co-researchers, such as attending conferences and educational events, which, in turn, helped build capacity. Overall, we recommend future groups conducting virtual CBPR to consider everyone’s comfort level and interest with transitioning to in-person activities, as existing approaches to build rapport and engagement are effective and may be preferred over the challenges that may arise from a hybrid set-up and during the initial adjustment period after transitioning to in-person meetings. After moving in person, seven co-researchers continued to regularly attend meetings and we anticipate the in-person move will strengthen our future co-design and implementation of a participatory research project.

## Conclusion

We successfully convened a CBPR group virtually during the COVID-19 pandemic. Co-researchers rated their priorities for what would make it easier to manage diabetes in the setting of homelessness. The nominal group technique we employed yielded the finding that the group prioritized addressing diabetes-related stigma through enhanced diabetes awareness.

While challenges abound in conducing CBPR virtually, our engagement strategy enabled this process to achieve the group’s objectives. These strategies included: providing the technology required and the IT support to minimize problems; engaging a peer researcher to help drive engagement; providing opportunities for socialization;and being responsive to co-researcher suggestions. While our process was successful in accomplishing what we set out to do, our group efficiency has improved significantly since we have been able to move to in person committee meetings. One additional facilitator for our group was the fact that not many of the co-researchers were actively experiencing absolute homelessness, with most having been housed prior to participating in our group process.

It is important to consider that these reflections may not be representative of other populations who may face different resource constraints due to varying socioeconomic positions, geographical considerations (e.g., communities spanning a country or multiple countries), or social roles (e.g., caregivers and students). For example, online meetings for CBPR may address barriers caregivers face in attending in-person meetings that require significant time commitments and travel [5]. Additionally, CBPR projects involving work with communities that span larger geographical locations (i.e., cross-country) may prefer online engagement compared to time- and resource-intensive in-person meetings [39]. However, based on our experience it is feasible to conduct CBPR virtually during pandemic times, yet, our group has found that resuming in-person interactions has been tremendously beneficial for developing rapport, sustaining interest and engagement, and maximizing productivity.

## Supplementary Information

**Additional file 1**. Semi-structured interview guide.

## Data Availability

Not applicable.
