# Supplementary material for: Lessons learned from a virtual Community-Based Participatory Research project: prioritizing needs of people who have diabetes and experiences of homelessness to co-design a participatory action project
Source: Res Involv Engagem. 2023 Jul 4;9:46. doi: 10.1186/s40900-023-00456-z (PMC10320889; doi:10.1186/s40900-023-00456-z)
Supplement: Supplementary file 1 — Additional file 1. Semi-structured interview guide. [file 40900_2023_456_MOESM1_ESM.docx]

**Appendices**

**Appendix A: Interview Guide for Initial Committee Member Interview**

Diabetes

1. Could you please tell us about yourself and what diabetes means in your life?

- How were you originally diagnosed with diabetes?
- How is your diabetes treated?
- How do you think your diabetes is controlled?
- Is there anything that worries you about diabetes?

Homelessness

1. What experience do you have with homelessness?

- When?
- How long?
- Why?
- Where did you tend to sleep during that time?
- How did you exit homelessness/get housed?

Diabetes + Homelessness

1. What was it like for you to be homeless and have diabetes?
2. How is this different from others who are homeless (but don’t have diabetes?)
3. How is this different from others who have diabetes (but aren’t homeless?)

Challenges

1. With regards to diabetes, what were some of the challenges that you faced at that time due to your housing status?
2. Could you tell us about some of the struggles that people might have when trying to manage diabetes during homelessness?

- Healthy food
- Medication access
- Testing supplies
- Physical activity
- Insulin & testing supply storage
- Transportation to appointments
- Relevance and applicability of information & education
- Priorities
- Chronic stress

Services

1. What are some of the healthcare services that you accessed at that time for diabetes care?

- Hospitals?
- DECs/DEPs?
- Family doctors?
- CHCs?
- In-shelter clinics/care?

1. Tell us a bit about your experiences there:

- Positive? Negative?
- What were some of the problems with the care you received there?
- What was excellent about the care you received there?
- What could be done differently to better meet your needs and those of people like you?

1. Did you access any other services that you think helped with your diabetes?

- Food: soup kitchens/community gardens
- Social workers
- Gym passes/physical activity programs

1. In your experience, what is lacking for homeless people that have diabetes? What are their needs that are currently not being met by the system?

Direction: This committee is committed to working with you on issues that are of importance and relevance to you specifically.

1. Could you tell us about what you think the group should be exploring?
2. Are there any areas of research that you think need to be explored?
3. What are you hoping to get out of your participation in this committee?

- What would you like to learn?

1. How can we be of service to the community of individuals who experience homelessness who also have diabetes?

- How would you like to help this community?
